# Supplementary material for: A Genetic Screen to Identify New Molecular Players Involved in Photoprotection qH in Arabidopsis thaliana
Source: Plants (Basel). 2020 Nov 13;9(11):1565. doi: 10.3390/plants9111565 (PMC7696684; doi:10.3390/plants9111565)
Supplement: Supplementary file 1 [file plants-09-01565-s001.zip › supplementale figure_Bru et al.docx]

| Low NPQ | | | | | | High NPQ | | | | Similar NPQ | |
| --- | --- | --- | --- | --- | --- | --- | --- | --- | --- | --- | --- |
| Normal green | | | Pale green | | | Normal green | | | Pale green | Normal green | Pale green |
| Normal F_v_/F_m_ | Low F_m_, F_o_ | High Fo | Low F_m_, F_o_ | High Fo | | Normal F_v_/F_m_ | Low F_m_, F_o_ | High F_o_ | Low F_m_, F_o_ | High F_o_ | Low F_m_, F_o_ |
|  |  |  |  | Lower Fv/F_m_ | Low Fv/F_m_ |  |  |  |  | Relax fast | Relax fast |
| *lcnp* (A205) | *roqh1* (A164) | A39 | *chlorina* (A26) | A37 | A73 | D101 | C4 | A76 | D73 | A232 | D48 |
| *lcnp* (A252) | *roqh1* (A108) | A36 | *chlorina* (A42) | A245 | A47 | A121 | D27 |  | A251 | A271 | D79 |
| A235 | A261 | BRT 162 | BRT 652 |  | A66 | D28 | D65 |  | D24 | A174 | D2 |
| A95 |  | E13 |  |  | A208 | D110 | BRT 1431 |  | D98 | BRT 1632 |  |
| D34 |  | A16 |  |  | E18 | D112 | BRT 853 |  | E40 |  |  |
| A171 |  | A57 |  |  | A9 | D128 |  |  | D83 |  |  |
| A244 |  | A40 |  |  | A61 | D53 |  |  | D132 |  |  |
| BRT 241 |  |  |  |  | A176 | C18 |  |  |  |  |  |
|  |  |  |  |  | C3 |  |  |  |  |  |  |
|  |  |  |  |  | E4 |  |  |  |  |  |  |
|  |  |  |  |  | E15 |  |  |  |  |  |  |
|  |  |  |  |  | BRT 511 |  |  |  |  |  |  |
|  |  |  |  |  | BRT 661 |  |  |  |  |  |  |
|  |  |  |  |  | BRT 1111 |  |  |  |  |  |  |

**Table S1. Mutants classification per phenotype.**

Mutant names highlighted in yellow correspond to the mutants presented in this study. For a given phenotype, only mutants coming from separate pools are listed.

**Table S2. Photosynthesis parameters F_o_, F_m_ and F_v_/F_m_.**

Data represent means ± SD (*n*= 3 individuals and 2 measures per individuals) shown in Figure 3B, 4B and 5B.

| Lines | F_o_ | F_m_ | F_v_/F_m_ |
| --- | --- | --- | --- |
| *soq1 npq4 gl1* | 108 $\pm$ 6 | 509 $\pm$ 20 | 0.79 $\pm$ 0.01 |
| *No.36* | 257 $\pm$ 13 | 502 $\pm$ 11 | 0.49 $\pm$ 0.03 |
| *No.39* | 264 $\pm$ 8 | 499 $\pm$ 11 | 0.47 $\pm$ 0.01 |
| *soq1 npq4 gl1* | 100 $\pm$ 6 | 482 $\pm$ 25 | 0.79 $\pm$ 0.00 |
| *No.37* | 186 $\pm$ 7 | 409 $\pm$ 15 | 0.55 $\pm$ 0.01 |
| *No.245* | 172 $\pm$ 10 | 422 $\pm$ 17 | 0.59 $\pm$ 0.02 |
| *soq1 npq4 gl1* | 112 $\pm$ 2 | 552 $\pm$ 10 | 0.80 $\pm$ 0.00 |
| *No.73* | 100 $\pm$ 8 | 490 $\pm$ 34 | 0.79 $\pm$ 0.03 |
| *No.251* | 96 $\pm$ 10 | 497 $\pm$ 33 | 0.80 $\pm$ 0.01 |


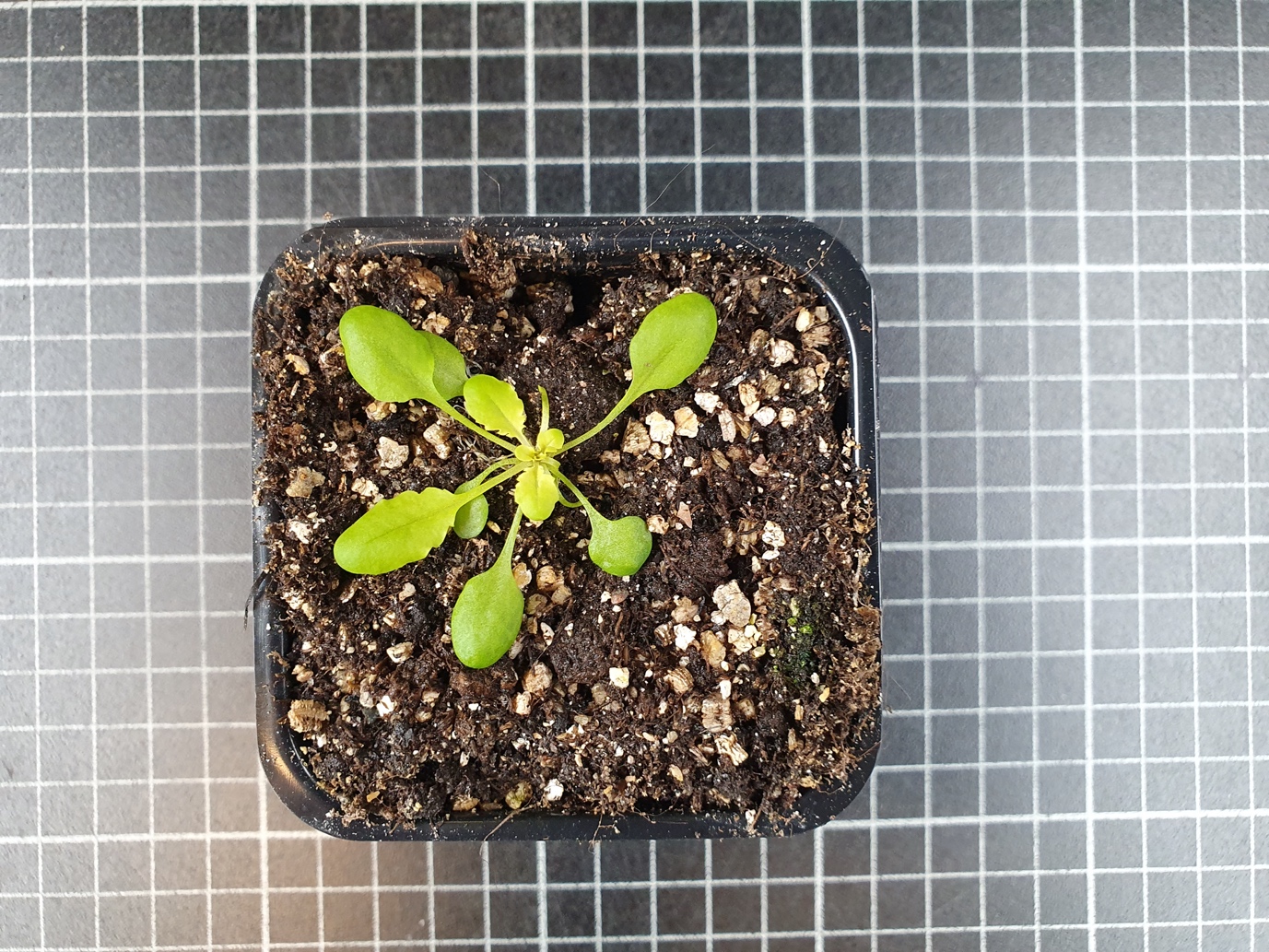


1
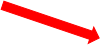


2
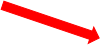


**Figure S1. Image of *No.251* mutant whole plant.**

Young leaves (1) display a more drastic pale green phenotype compared to older leaves (2)**.**
